# Mycobacterial PE/PPE Proteins at the Host-Pathogen Interface

**DOI:** 10.1155/2011/497203

**Published:** 2011-01-26

**Authors:** Samantha L. Sampson

**Affiliations:** Section of Microbiology, Centre for Respiratory Infection, Imperial College London, Armstrong Road, London SW7 2AZ, UK

## Abstract

The mycobacterial PE/PPE proteins have attracted much interest since their formal identification just over a decade ago. It has been widely speculated that these proteins may play a role in evasion of host immune responses, possibly via antigenic variation. Although a cohesive understanding of their function(s) has yet to be established, emerging data increasingly supports a role for the PE/PPE proteins at multiple levels of the infectious process. This paper will delineate salient features of the families revealed by comparative genomics, bioinformatic analyses and genome-wide screening approaches and will summarise existing knowledge of subcellular localization, secretion pathways, and protein structure. These characteristics will be considered in light of findings on innate and adaptive host responses to PE/PPE proteins, and we will review the increasing body of data on B and T cell recognition of these proteins. Finally, we will consider how current knowledge and future explorations may contribute to a more comprehensive understanding of these intriguing proteins and their involvement in host pathogen interactions. Ultimately this information could underpin future intervention strategies, for example, in the area of new and improved diagnostic tools and vaccine candidates.

## 1. Introduction

Tuberculosis (TB) represents an ongoing threat to global health, with the current epidemic fuelled by HIV-coinfection and an increasing incidence of drug resistant strains of *Mycobacterium tuberculosis * [1]. Effective new interventions are urgently needed, and genes that are unique to mycobacteria may provide a starting point for developing these. The intriguing *pe/ppe* genes first attracted attention due to their genetically hypervariable nature [2, 3] and were initially exploited as informative molecular markers for mycobacterial strain typing [2, 4]. Shortly thereafter, the first *M. tuberculosis* genome sequence was completed, and it was revealed that these variable regions were in fact part of two extensive families encoding almost 200 putative proteins [5]. It is now known that these genes are unique to mycobacteria and are particularly abundant in pathogenic mycobacteria, such as *M. tuberculosis*. Naturally, the PE/PPE families have provoked much speculation, although we have yet to establish a complete understanding of their function. However, the advent of the mycobacterial genomic age, together with improved molecular tools and a deeper understanding of the immunopathogenesis of TB, has advanced our knowledge of these gene families and the potential functions of their encoded proteins.

## 2. PE/PPE Genomics

**Figure 1:**
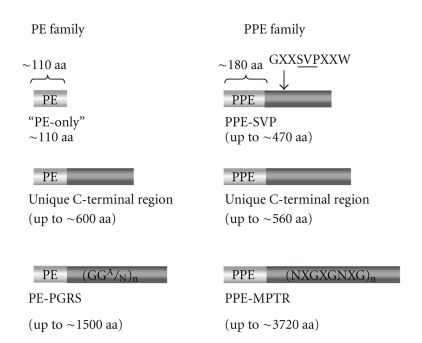
Schematic representation of PE and PPE family subgroups. PE and PPE proteins possess relatively conserved N-terminal domains of approximately 110 aa and 180 aa, respectively. One subgroup of the PPE family incorporates a characteristic “SVP” motif at approximately 350 aa. Both PE and PPE families can be divided into distinct subgroups on the basis of their variable C-terminal domains. The regions encoded by the Polymorphic GC-Rich Sequence (PGRS) of the *pe* family and the Major Polymorphic Tandem Repeats (MPTR) of the *ppe* family are major contributors to genome polymorphism.

Analysis of the *M. tuberculosis* H37Rv genome sequence revealed the presence of two novel gene families that comprise almost 10% of the coding capacity of the genome [5]. These were designated the *pe *and *ppe* genes, after highly conserved Proline-Glutamate and Proline-Proline-Glutamate residues near the start of their encoded proteins. The proteins can be categorized into subgroups, encompassing members with highly variable length and sequence features (Figure 1) [5]. The relatively conserved N-terminal is approximately 110 amino acids (aa) and 180 aa in the PE and PPE families, respectively. The smallest members of both families consist of just this conserved domain, while other subclasses have additional C-terminal regions. The PE_PGRS (polymorphic GC-rich sequence) and PPE_MPTR (major polymorphic tandem repeat) subgroups possess C-terminal regions of enormously variable size—these can reach over 3700 aa in length; they are also the family members which exhibit the most sequence variation. 

The remarkable length and extensive sequence variation of the PE_PGRS and PPE_MPTR proteins appear to be primarily associated with lengthy stretches of GC-rich, imperfect triplet repeats within their associated genes. These are thought to be hotspots for recombination events and other mutations, including insertion of transposable elements [5, 6]. Other *pe/ppe*-associated sequence variation includes large sequence polymorphisms which appear to be mediated by highly homologous sequences in the conserved 5′ regions of *pe/ppe *genes [6]. Given the diverse mechanisms whereby *pe/ppe *polymorphisms can arise, together with the high potential for redundancy within the gene families, it is perhaps unsurprising that some *pe/ppe *genes vary extensively in clinical isolates [7–9]. Consequently, it has been speculated that these proteins represent a source of antigenic variation which allows the organism to escape antigen-specific host responses [5, 7, 8]. However, this hypothesis is as yet unproven, and there is little evidence to support rapid within-host diversification of these genes. Therefore if the observed sequence variation is providing a source of antigenic diversity, the benefits of this are likely to operate on a population-wide scale, rather than within individual hosts. One study has demonstrated a moderately significant association of large sequence variation in *pe_pgrs33* with noncavitary TB and case clustering [10]. However, further experimentation will be required to establish whether there is a causal link between these observations, and if so, to determine the underlying mechanistic basis. It is worth noting that while the *pe_pgrs* and *ppe_mptr *genes represent some of the most variable regions of the *M. tuberculosis *chromosome, it is an oversimplification to extend this to all *pe/ppe* family members, as some are in fact conserved across strains and species [11]. It is important to bear this distinction in mind when considering potential functional roles and to extrapolate experimental results with caution. 

An increasing wealth of mycobacterial genome sequence data has advanced our knowledge of the sequence diversity and evolutionary history of the *pe/ppe* gene families. One comparative genomics study revealed that the evolution and major expansion of the *pe/ppe* families is closely associated with the *esx* regions [12]. These encode the so-called Type VII or ESX secretion systems, of which there are 5 in *M. tuberculosis * [13]. The best characterized of these is ESX-1, which has been implicated in mycobacterial virulence through the secretion of effectors such as EsxA and EsxB [14–16] (otherwise known as ESAT-6 (early secreted antigenic target of 6 kDa) and CFP-10 (culture filtrate protein of 10 kDa), resp.). Gey van Pittius and colleagues further showed that the most repetitive and variable family members represented by the PE_PGRS and PPE_MPTR subgroups are also the most phylogenetically recent. These subgroups are restricted to the pathogenic mycobacteria, and their massive expansion is closely associated with the most recently duplicated *esx *gene cluster, *esx-5* [12]. These findings were suggestive of a functional link between the PE/PPE families and the ESX secretion systems. Indeed, it has since been demonstrated that the secretion of multiple PE/PPE family members is ESX-mediated [17–19].

## 3. Transcriptomics, Proteomics, Structure, and Subcellular Location

In tandem with burgeoning mycobacterial genome data, other “-omics” undertakings have shed light on certain aspects of the PE/PPE families. An exhaustive account of *pe/ppe *transcriptomics is beyond the scope of this paper. However, it is worth mentioning that a study of *M. tuberculosis pe/ppe *gene expression under 15 different conditions revealed that 128/169 *pe/ppe *genes analysed were differentially regulated [20]. Other groups have demonstrated that several *pe/ppe *genes are upregulated upon macrophage infection and in host tissues [21–24]. For example, Rachman et al. demonstrated that two *pe *genes (*pe11, pe34*), four* pe_pgrs (pe_pgrs14, 33, 55 *and* 57), *and three *ppe-mptr *genes (*pe_pgrs54, 55 and 62) *were all upregulated in human lung granulomas compared to *in vitro* grown bacteria [24]. Together with other published expression data, these results lend support to the idea that the *pe/ppe* genes play important functional roles *in vivo*. 

Characterisation of regulatory and other mutants has revealed that subsets of *pe/ppe* genes are frequently among the genes whose expression levels are altered [25–27]. In one example, disruption of the virulence-associated PhoPR two-component regulator resulted in altered expression of at least 14 *pe/ppe *genes. These include *pe_pgrs41*, which demonstrated a striking 143-fold higher expression level in wild type *M. tuberculosis* compared to the *phoP *mutant [26]. It has been demonstrated that selected *pe/ppe* genes may be differentially regulated in genotypically diverse clinical isolates [28–30]. For instance, Gao et al. examined gene expression in 10 clinical isolates of *M. tuberculosis* and found that 28 of the 77 *pe/ppe* genes included in the analysis showed variable expression [29]. There is also evidence for divergent regulation of specific family members within individual strains [31, 32]. This is exemplified by *pe_pgrs16 *and *pe_pgrs26*, which are thought to be inversely regulated *in vitro* [31], although the mechanistic basis for this apparently coordinated expression is unclear. Taken together, available data does not support global regulation of *pe/ppe* gene expression, suggesting that there is likely to be a relatively high degree of plasticity in the *pe/ppe* expression repertoire. Although this may suggest that some *pe/ppe *genes are not required at all, this could also indicate that members within different subgroups of the family play subtly different functional roles and are required at different stages of disease. 

The existence of multiple highly similar *pe/ppe* family members suggests a high potential for functional redundancy within subgroups. It is therefore not surprising that very few of these genes are essential for *in vitro* or *in vivo* growth, as demonstrated in a series of studies by Rubin and colleagues [33–35], Table S1 (see Table S1 in supplementary material available online at doi:10.1155/2011/497203). This high degree of redundancy contributes to one of the experimental challenges presented by this family, that is, that mutations in single *pe/ppe* genes may lack measurable phenotypes. However, there are some examples where this is not the case, and analysis of selected *pe/ppe* mutants has yielded important functional insights, as will be described below.

High-throughput proteomics-based studies have highlighted further challenges of working with PE/PPE proteins. The proteins are often very large, may be tightly associated with the cell wall, and can have limited proteolytic cleavage sites. These inherent properties may restrict the utility of some high-throughput proteomics approaches [18]. Nonetheless, using optimized growth conditions and sample preparation methods in conjunction with sensitive detection methodologies, some studies have successfully identified the subcellular localization of selected PE/PPE proteins in different mycobacterial strains and species (Table S1). For example, using proteolytic shaving of intact *Mycobacterium avium subsp. paratuberculosis *followed by LC-MS/MS, Newton et al. identified 2 PPE orthologues associated with the cell wall [36]. PE_PGRS and PPE orthologues were also identified in the cell membrane fraction of *Mycobacterium immunogenum * [37], and a recent detailed analysis of the *Mycobacterium marinum* capsule using cryoelectron microscopy in conjunction with LC-MS/MS demonstrated that 6/25 major cell surface proteins were members of the PE/PPE families [38]. Similarly, Målen et al. utilized MALDI-MS and LC-MS/MS to identify at least 7 and 16 PE/PPEs in the *M. tuberculosis *culture filtrate and envelope fractions, respectively [39, 40]. High-throughput proteomics approaches therefore suggest that cell wall/surface localization is a characteristic of several PE/PPE proteins. This has been corroborated by more focused studies which provide further substantial evidence indicating that numerous PE/PPE family members are associated with the bacterial cell wall [37, 38, 40–43]. These include PPE36 [42], PPE68 [41], PE_PGRS33 [44], PE_PGRS63 [43], and PPE_MPTR34, with further examples listed in Table S1. In some cases, results indicate surface exposure of these proteins [17, 44–47]. Although it has been proposed that PE/PPE proteins could form complex surface structures [48], this hypothesis has yet to be addressed experimentally.

To date, only one PE/PPE protein structure has been solved, perhaps reflecting the difficulty experienced with recovering stable, soluble recombinant PE/PPE proteins. Strategies used to successfully overcome this challenge include on-column refolding [49], use of mycobacterial host strains for expression [50], and coexpression of cognate PE/PPE pairs [51, 52]. Using the latter approach, Strong and coworkers successfully purified recombinant PE25/PPE41 and subsequently determined the crystal structure, which showed that the protein pair forms a stable 1 : 1 heterodimer [52]. The PE/PPE complex is reminiscent of the EsxA/EsxB complex, which could suggest common secretion pathways. Intriguingly, the PE25/PPE41 complex displays an apolar stripe on one face, which could represent a docking site for an as-yet unidentified bacterial or host target [52]. An attempted structure/function analysis revealed that the heterodimeric complex shares some features of signal transduction molecules [52], although this has yet to be explored experimentally. It is unknown whether the PE25/PPE41 structure is truly representative of the rest of the protein family. Indeed, it is unlikely that this structure can be extrapolated to family members which are not predicted to form heterodimers [53] or which demonstrate substantial variation in sequence length and content. The structural biology of PE/PPE proteins is therefore an area in much need of further development, as characterization and comparative analysis of additional PE/PPE structures could perhaps provide clues to their possible function. Another crucial step towards determining the function of the PE/PPE proteins will be identifying their interaction partners. In particular, determining host targets of these proteins could provide critical insights into their impact on host responses. 

As mentioned above, the PE/PPE proteins are intimately associated with the ESX systems [12]. A functional link has been elucidated by the work of Abdallah and colleagues, who demonstrated that the ESX-5 apparatus mediates the secretion of multiple PE/PPE proteins, including members of the PE_PGRS and PPE_MPTR subsets [17, 18]. The ESX-5 locus is implicated in *M. marinum* virulence [17, 54], although it remains to be seen whether the secreted PE/PPE proteins play a central role in this. In addition, ESX-5-mediated PE/PPE secretion has yet to be demonstrated for *M. tuberculosis*. Of course, given the parallels with the EsxA/EsxB complex and the close association with the ESX secretion apparatus, it is tempting to speculate that PE/PPE complexes (or the individual constituent proteins) could be virulence effectors secreted by ESX-5. Once again, caution should be exercised when extrapolating these results to other family members; it is worth noting that some PE/PPE proteins in fact possess functional N-terminal signal peptide cleavage sites [39]. It is therefore plausible that their secretion/localization may occur via other mechanisms, for example, the Sec-dependent export pathway. In support of this, several studies have reported cell-wall localization of PE/PPE proteins in *Mycobacterium smegmatis, *which lacks the ESX-5 region [44, 46, 47, 55]. In addition to the possible role of ESX-5, there is some evidence implicating ESX-1 in PE/PPE secretion. For example, the genes encoding PE35/PPE68 are situated within the *esx-1* region, and one study has suggested that PE35 secretion is ESX-1-dependent [19]. Others have demonstrated cell-wall association of PPE68 in *M. tuberculosis* [40, 41], and this protein also interacts with multiple components of the ESX-1 machinery [56, 57]. Interestingly, disruption of *ppe68* is associated with increased secretion of ESAT-6, leading to the suggestion that PPE68 may act as an ESX-1 gating protein [58], although this has yet to be experimentally verified. 

Regardless of their secretion mechanism, several lines of evidence support surface localization of PE/PPE proteins (Table S1). Some PE/PPEs may be actively secreted or passively shed from the bacterium into the host cell milieu, and could even be released from host cells via exosomes [59]. Thus, they are ideally positioned to interact with the host immune system. Indeed, there is extensive evidence that they do so in ways that could have important consequences for the outcome of infection.

## 4. PE/PPEs Modulate Innate Immune Responses

There is mounting evidence that PE/PPE proteins interact with host components and thereby modulate and possibly subvert critical innate immune pathways. Ramakrishnan et al. provided the first definitive evidence that (at least some) PE_PGRS proteins are virulence factors, when they showed that two *M. marinum pe_pgrs* genes were required for survival in macrophages and granulomas [60]. Since then, the most extensively studied “examplar” member of the *M. tuberculosis* PE_PGRS family, namely PE_PGRS33, has provided some insight into the numerous ways in which these proteins could modulate host immune responses. Detailed examination revealed that PE_PGRS33 interacts directly with TLR-2, thereby mediating apoptosis and cytokine secretion [61]. Others have confirmed that PE_PGRS33 promotes host cell apoptosis [59], and, in one study, necrosis [62]. Interestingly, sequence variants of PE_PGRS33 and other PE_PGRS family members elicited differential effects [61]. PE_PGRS11 and PE_PGRS17 were also shown to interact with TLR-2, prompting maturation and activation of dendritic cells [63]. Independent studies have subsequently shown that PPE18 and an MPTR-containing domain of PPE_MPTR34 can also modulate host responses through TLR-2 [64, 65]. Although it has yet to be established if TLR-2 engagement is a common property of PE/PPE proteins, these results suggest that the additive host response to interaction with multiple PE/PPE proteins may be very complex, and could profoundly impact on the course of disease. 

PE/PPE proteins can also influence macrophage function by other means. For example a BCG *pe_pgrs33 *transposon mutant demonstrated reduced entry into macrophages [66], suggesting that PE_PGRS33 may play a role in promoting macrophage uptake. In contrast to other bacteria, this may be advantageous for *M. tuberculosis *at the very early stage of infection, as it could promote dissemination and colonization of multiple organs. This strategy is only effective because the pathogen has evolved multiple strategies to overcome the hostile environment encountered within host cells. In this context, several PE/PPE proteins have been shown to facilitate, or be required for, *in vivo* survival [35, 60, 67, 68]. A key macrophage defence mechanism is phagosome maturation and acidification, which normally results in killing of intraphagosomal pathogens. However, *M. tuberculosis* is adept at subverting this pathway, and there is evidence that different PE/PPE family members may contribute to this. Selected PE/PPE family members have been implicated in the modulation of vacuole acidification [69–71]. For example, a high-throughput analysis of a *Mycobacterium bovis *BCG tranposon mutant library identified four *pe_pgrs *mutants (*pe_pgrs5, 28, 44, 59*) and three *ppe_mptr* mutants (*ppe_mptr10, 16, 21*) that were enriched in acidified phagosomes. A more recent study showed that a *M. tuberculosis ppe54* transposon mutant was impaired in its ability to arrest phagosome maturation and trafficked rapidly into acidified compartments [72]. 

Available data therefore indicates that selected PE/PPE family members play important roles in subverting innate immune responses, and together with other bacterial mediators, may assist the pathogen in establishing itself within the host. Once this has occurred, PE/PPEs may then contribute to processes which allow the pathogen to persist within host tissues. Intriguingly, enzymatic functions have been assigned to 3 PE proteins: PE_PGRS11 has been reported to be a functional phosphoglycerate mutase [55], while both PE_PGRS63 (LipY, [43]) and PE11 (LipX, [73]) demonstrate lipase activity. The latter 2 proteins may therefore play a role in energy provision. It should be noted though that these are exceptional examples; they appear to have arisen as a result of recombination events which lead to functional divergence, and enzymatic activity is not predicted to be a general feature of the PE/PPE proteins. 

Once established within their intracellular niche, there are a number of ways in which mycobacteria can limit engagement of the adaptive immune response, and the PE/PPE proteins may contribute to some of these mechanisms. The observation that PE_PGRS proteins bear some resemblance to the Epstein-Barr virus nuclear antigen (EBVNA) gave rise to speculation that, akin to the EBVNA, the PE_PGRS proteins may inhibit antigen processing [5]. This has subsequently been demonstrated for PE_PGRS33 [48] and PE_PGRS17 [74], supporting the idea that these proteins may assist in immune evasion by limiting antigen presentation, thereby preventing recognition and killing of mycobacteria-infected host cells.

Taken together, the observations described above suggest that different PE/PPE proteins may play distinct, but complementary roles as the infection progresses, acting in concert to facilitate adaptation to the hostile host environment. Members of these families may be critical mediators of host responses which ultimately determine the outcome of infection. Understanding their individual and cumulative impact on host responses is an important undertaking which could provide the starting point for novel interventions. The large number and diverse effects of these proteins present some experimental challenges. Future explorations may therefore benefit from systems-level approaches to help unravel their biological role.

## 5. B and T Cell Recognition of PE/PPE Proteins

### 5.1. Humoral Immunity

The contribution of humoral responses to controlling *M. tuberculosis *infection has long been underappreciated. However, it is known that mycobacteria-specific antibodies can both influence mycobacterial dissemination and modulate potentially detrimental inflammatory tissue responses [75, 76]. It is increasingly recognized that B cells can exert an influence on T cells [77, 78] and are an important constituent of granuloma architecture [79]. B cells are thus likely to be more important in determining the outcome of infection with *M. tuberculosis* than previously supposed, and ignoring this aspect of the host immune response may restrict our understanding of TB immunopathology. In this context, PE/PPE family members are a potentially rich source of B cell epitopes, and a number of PE/PPE proteins may be surface exposed, increasing the likelihood that they could be targeted by the humoral response. Accordingly, numerous PE/PPE proteins have been shown to elicit B cell responses (Table S1). There is evidence for both members of the PE_PGRS and PPE_MPTR families that the highly repetitive domains are chiefly responsible for eliciting antibody responses. Delogu and Brennan were the first to demonstrate differential B and T cell targeting of the conserved N-terminal and highly repetitive, more variable, C-terminal of PE_PGRS33; DNA vaccination of mice with only the PE domain elicited predominantly cell-mediated immunity and subsequent protection against challenge, whereas a full-length PE_PGRS33 construct promoted a nonprotective, B cell-skewed response [80]. In agreement with these results, PPE_MPTR42 was found to elicit a primarily humoral response, directed toward the glycine and asparagine-rich repeat domain [81]. In other work, it was shown that the full-length PE_PGRS17 and PE_PGRS62 proteins were preferentially recognized in comparison to the PE-only versions [82]. Further elucidation of differential immune targeting of PE/PPE proteins could enable fine-tuning of new vaccine candidates.

### 5.2. PE/PPE Proteins as T Cell Immunogens

Cell-mediated immunity is especially important in the control of *M. tuberculosis *infection [83, 84]. Therefore, T cell antigens are of great interest for vaccine development and could form useful components of subunit DNA or protein vaccines or engineered into live recombinant vectors. Consequently, numerous studies have investigated the ability of PE/PPE proteins to elicit T cell responses (Table S1). For example, T cell expression cloning was successfully exploited to identify T cell immunogens in *M. tuberculosis*-sensitized individuals [85]; this relatively high-throughput approach resulted in the identification of the potent T cell antigen Mtb39a, which is encoded by *Rv1196*/*ppe18*. In subsequent work, this antigen has been evaluated as part of the polyprotein subunit vaccine candidate, Mtb72f, as described further below (Section 6.2). In addition to PPE18, at least 20 PE/PPE proteins have been reported to elicit CD4 and/or CD8 responses, either in the form of whole recombinant proteins or as individual peptides (Table S1). Together, these results suggest that PE/PPE proteins are worthy of further evaluation as potentially protective antigens for inclusion in new TB vaccine candidates.

Aside from the possible practical applications of PE/PPE-derived antigens (see Section 6), their high degree of immunogenicity could also provide some clues regarding their biological function. A recent study which combined multiple genome sequence comparisons with analysis of published immunogenicity data reported an unexpectedly high level of conservation of human T cell epitopes [86]. This runs counter to the existing dogma that antigenic targets will exhibit a relatively high degree of variation due to selection pressures imposed by host immune responses. Unfortunately, the high-throughput sequencing methodology used in the study imposes inherent technical limitations, which impact substantially on *pe/ppe* genes; due to the short sequence reads, highly repetitive, multicopy sequences are difficult to accurately assemble. As a result, PE/PPE proteins were excluded from their analysis. It is possible that some members of these protein families, in particular within the PE_PGRS and PPE_MPTR subgroups, could be exceptions to the described finding. However, our own recent work suggests that a large subset of the PE/PPE family, which is relatively well conserved across multiple clinical isolates, is also highly immunogenic (manuscript in preparation). The most conserved regions of the proteins are also the most well recognized, and even when PE/PPE amino acid sequences do vary, this does not necessarily impair epitope recognition by cross-reactive T cells (manuscript in preparation). Taken together, this suggests that at least some PE/PPE family members will fit the paradigm revealed by Comas et al. [86]. This raises the question of whether the high level of immunogenicity displayed by proteins such as the PE/PPEs confers a selective advantage on the pathogen. As speculated by others [87], it is possible that robust inflammatory responses lead to lung damage which could promote *M. tuberculosis* transmission. An alternative hypothesis is that these potent T cell antigens could provide a further means of immune evasion by overwhelming and misdirecting the adaptive immune response. 

To date, there is little experimental data to support the contention that PE/PPE proteins contribute to antigenic variation in the conventional understanding of the phenomenon. However, there is substantial evidence that these proteins contribute to immune evasion via other important mechanisms, as described above.

## 6. Translational Applications

The findings described in the preceding sections have implications for both vaccine development and diagnostic tools. 

### 6.1. Diagnostic Tools

In terms of new diagnostic tools, several highly immunogenic PE/PPEs have been identified, which could contribute to the development of antigen cocktails to be used in IFN-*γ* release assays or antibody-based tests. However, there are some caveats. Firstly, there is the issue of intraspecies cross-reactivity—a number of PE/PPE proteins are present in mycobacteria outside of the *M. tuberculosis *complex [12] and are recognised immune targets [36, 81, 88, 89]. Therefore, antigens would need to be rigorously evaluated to ensure species-specificity. Secondly, although there are some indications that some PE/PPE-derived antigens may distinguish between vaccinated and nonvaccinated individuals, or between different forms of disease and/or stages of infection, [55, 82, 90–95], existing data suggests that differential diagnostic capability does not apply to all PE/PPE proteins [90, 91, 96]. Therefore their utility in distinguishing between active disease, latent infection and vaccinated individuals will likely be limited to selected family members, and would need rigorous validation in larger patient cohorts prior to clinical implementation.

### 6.2. Vaccines

PE/PPE proteins are a potentially rich source of T-cell antigens, and several of these have been assessed for possible inclusion in new vaccines [80, 97–102], Table S1. As mentioned above, the PPE18-based subunit vaccine candidate Mtb72f, formulated with the adjuvant AS02A, has yielded promising results in multiple animal models [103, 104] and is now undergoing Phase I clinical trials [105, 106]. Similarly, evaluation of 2 polyprotein vaccine candidates which incorporate PPE_MPTR42 is at a relatively advanced stage. PPE_MPTR42 was shown to confer partial protection in mice when formulated with the TLR-9 agonist CpG [97]. The fusion protein ID83 (which incorporates Rv1813, Rv3620, and PPE_MPTR42) elicits protective immunity in mice [97], which can be modulated by use of different adjuvants and/or route of immunization [107]. In recent work, a similar fusion protein (ID93, which incorporates PPE_MPTR42, Rv1813, Rv3620 as well as a fourth antigen, Rv3619) has been shown to elicit protection against challenge with virulent *M. tuberculosis *in both mice and guinea pigs [108]. As it is likely that subunit vaccine candidates (either DNA or protein-based) will be introduced in the context of a BCG-prime/subunit boost regimen, it is appropriate that these be tested in this setting; importantly, ID93 offers enhanced protection in a BCG-prime/ID93-boost regimen in guinea pigs [108]. Furthermore, ID93 is immunogenic in cynomolgous macaques and elicits polyfunctional CD4 and CD8 responses in peripheral blood mononuclear cells from BCG-vaccinated humans [108]. Together, these promising preclinical results support further evaluation of ID93 in humans in Phase I clinical trials.

The protective capacity of additional PE/PPE-derived subunit candidates is also under investigation. For example, DNA vaccines based on PPE44 [99], PPE41 [100], and the PE domain of PE_PGRS33 [80] have all been shown to elicit protection in mice. In addition, PE_PGRS62- and PE20-derived DNA vaccines have been shown to reduce guinea pig bacterial lung burden by >0.5 log [101]. An alternative to the BCG prime/subunit boost approach is to heterologously express candidate antigens in a live vaccine vehicle, and thereby appropriately supplement/modulate immune responses. Wang et al. have explored this approach by coexpressing PPE57 and Ag85B in BCG and have demonstrated that the recombinant BCG strain elicits enhanced immunogenicity in mice [102]. However, it has yet to be determined whether this approach will result in improved protection.

In addition to their immunogenicity, other features of PE/PPE proteins may be beneficial to vaccine development efforts. For example, the PE domain is thought to be responsible for protein localization to the cell wall [44], and this property could be exploited to ensure optimal localization of PE/PPE-derived or other heterelogous antigens in live recombinant vaccines. Secondly, selected PE/PPE proteins may be potent immune-modulators that could be exploited to fine-tune the immune response to enhance protective capabilities of vaccine antigens. 

Although PE/PPE proteins show potential for exploitation in vaccine development efforts, several issues need to be considered. Firstly, many of the known PE/PPE immunogens have been identified on the basis of their ability to stimulate IFN-*γ* production. While this cytokine is clearly important for the control of TB, it is not necessarily the best predictor of protection against subsequent challenge [109]. The simultaneous production of other cytokines, such as IL-2, has been shown to be a significant component of a protective immune response [110]. Likewise, the phenotypic makeup of the responding cell population is also important; for example, the presence of antigen-specific central memory T cells correlates with protection [111]. Future antigen discovery and evaluation exercises therefore need to consider a broader cytokine profile, as well as the phenotype of the responding cell populations. A second point of caution is that the many of the immunogenic PE/PPE epitopes which have been identified are recognized during active infection, suggesting that the ability to mount an immune response against them does not necessarily confer protection. However, with a more comprehensive understanding of what constitutes a protective immune response, it may be possible to fine-tune factors such as vaccination dose, timing, and formulation to accordingly modulate responses to vaccination. Finally, it will also be important to have a clearer understanding of PE/PPE function and possible adverse effects on host responses. For example, at least 3 PE_PGRS proteins, PPE_MPTR34, and PPE18 have all been shown to interact directly with surface receptors on host macrophages eliciting responses such as apoptosis and cytokine production [61, 63–65] that may not necessarily be desirable outcomes. Until we have a better understanding of the nuances of PE/PPE-host interactions, the use of these proteins as vaccine candidates should be cautiously approached. 

Importantly, consideration is increasingly being given to the “real-world” setting in which new TB vaccine candidates will be tested and utilized. For example, epitope prediction tools can be used to assess the prevalence of peptides which are likely to bind MHC alleles found in the target population [112]. Also, multiple genome sequence comparisons can help to determine the degree of conservation of predicted MHC binding peptides in circulating mycobacterial strains [113]. Using Mtb72f as an example, McNamara et al. applied a combination of epitope prediction software packages to demonstrate that several MHC DRB1 alleles would have limited binding to predicted PPE18 epitopes [114]. They further showed that predicted binding peptides were relatively unconserved [114], in accordance with an earlier report [113]. On the basis of these results, it was suggested that Mtb72f might have limited efficacy in selected populations [114]. Although epitope prediction tools are still in need of refinement, *in silico *approaches such as these can be applied to prioritize antigen screening. They could also be used to predict the likely efficacy of selected vaccine candidates prior to undertaking lengthy and resource-intensive clinical trials.

## 7. Conclusions

More than a decade after their discovery, a cohesive understanding of the function of the PE/PPE proteins remains elusive. The large and complex families pose a number of experimental challenges. However, some interesting themes are emerging: several members of the family trigger a range of innate immune responses, and many are targets of the adaptive immune system. These families are a potentially rich source of diagnostic and vaccine antigens and could even find application as immunomodulatory agents. However, it will be critical to improve our understanding of their function and potential effects of these proteins on the host. Fundamental areas in need of attention include PE/PPE structural biology, identification of PE/PPE host targets, and bacterial interaction partners and an expanded understanding of the mechanisms whereby these proteins modulate host immune responses. Some of the experimental challenges posed by these large and complex proteins families could be addressed using approaches informed by the field of systems biology. Future investigations which exploit the wealth of mycobacterial genome sequence data in the context of our increasing understanding of host and pathogen biology may provide a platform for answering some of the intriguing questions surrounding these enigmatic proteins.

## Supplementary Material

Table S1: PE/PPE families—Immune recognition and functional data. Summary of published data on mycobacterial pe/ppe genome arrangement, essentiality, subcellular localization, cellular and humoral responses along with other relevant functional.
